# Trends in mortality in septic patients according to the different organ failure during 15 years

**DOI:** 10.1186/s13054-022-04176-w

**Published:** 2022-10-03

**Authors:** Carolina Lorencio Cárdenas, Juan Carlos Yébenes, Emili Vela, Montserrat Clèries, Josep Mª Sirvent, Cristina Fuster-Bertolín, Clara Reina, Alejandro Rodríguez, Juan Carlos Ruiz-Rodríguez, Josep Trenado, Elisabeth Esteban Torné

**Affiliations:** 1 Intensive Care Department, Hospital Universitari Dr. Josep Trueta, Girona, Spain; 2 Intensive Care Department, Hospital de Mataró, Mataró, Spain; 3 Digitalization for the Sustainability of the Healthcare System (DS3), IDIBELL., Barcelona, Spain; 4 Intensive Care Department, Hospital Universitari Joan XXIII, Tarragona, Spain; 5 Intensive Care Department, Shock, Organ Dysfunction and Resuscitation Research Group, Vall d’Hebron, Vall d’Hebron Hospital Universitari, Vall d’Hebron Barcelona Hospital Campus, Barcelona, Spain; 6 Intensive Care Department, Hospital Mútua de Terrassa, Terrassa, Spain; 7 Pediatric Intensive Care Department, Hospital Universitari Sant Joan de Déu, Barcelona, Spain; 8 Àrea de Sistemes d’informació, Servei Català de la Salut (CatSalut), Barcelona, Spain; 9 Universitat de Girona. UdG., Girona, Spain

**Keywords:** Sepsis, Sepsis syndrome, Septic shock, Sepsis mortality, Epidemiologic methods, Sepsis epidemiology, Multiple organ dysfunction syndrome, Sequential organ failure assessment score, Sepsis-related organic failure

## Abstract

**Background:**

The incidence of sepsis can be estimated between 250 and 500 cases/100.000 people per year and is responsible for up to 6% of total hospital admissions. Identified as one of the most relevant global health problems, sepsis is the condition that generates the highest costs in the healthcare system. Important changes in the management of septic patients have been included in recent years; however, there is no information about how changes in the management of sepsis-associated organ failure have contributed to reduce mortality.

**Methods:**

A retrospective analysis was conducted from hospital discharge records from the Minimum Basic Data Set Acute-Care Hospitals (CMBD-HA in Catalan language) for the Catalan Health System (CatSalut). CMBD-HA is a mandatory population-based register of admissions to all public and private acute-care hospitals in Catalonia. Sepsis was defined by the presence of infection and at least one organ dysfunction. Patients hospitalized with sepsis were detected, according ICD-9-CM (since 2005 to 2017) and ICD-10-CM (2018 and 2019) codes used to identify acute organ dysfunction and infectious processes.

**Results:**

Of 11.916.974 discharges from all acute-care hospitals during the study period (2005–2019), 296.554 had sepsis (2.49%). The mean annual sepsis incidence in the population was 264.1 per 100.000 inhabitants/year, and it increased every year, going from 144.5 in 2005 to 410.1 in 2019. Multiorgan failure was present in 21.9% and bacteremia in 26.3% of cases. Renal was the most frequent organ failure (56.8%), followed by cardiovascular (24.2%). Hospital mortality during the study period was 19.5%, but decreases continuously from 25.7% in 2005 to 17.9% in 2019 (*p* < 0.0001). The most important reduction in mortality was observed in cases with cardiovascular failure (from 47.3% in 2005 to 31.2% in 2019) (*p* < 0.0001). In the same way, mean mortality related to renal and respiratory failure in sepsis was decreased in last years (*p* < 0.0001).

**Conclusions:**

The incidence of sepsis has been increasing in recent years in our country. However, hospital mortality has been significantly reduced. In septic patients, all organ failures except liver have shown a statistically significant reduction on associated mortality, with cardiovascular failure as the most relevant.

**Supplementary Information:**

The online version contains supplementary material available at 10.1186/s13054-022-04176-w.

## Background

Sepsis is defined as an organ dysfunction secondary to a dysregulated immune response to an infectious process [1]. Incidence can be estimated between 250 and 500 cases/100.000 people per year [2–7] and is responsible for up to 6% of total hospital admissions [4]; up to 50% can require ICU admission [4]. Incidence of community acquired sepsis that requires admission to ICU has been estimated to be 50 cases/100.000 inhabitants per year [8].

Identified as one of the most relevant global health problems, sepsis is the condition that generates the highest costs in the US healthcare system [5] and is responsible for practically half of hospital deaths [6], even above myocardial infarction and stroke [7]. Improvements in knowledge of the epidemiological behavior of sepsis could be useful for a better approach and management of this pathology.

Although the absolute values obtained from epidemiological studies may be biased by different selection procedures, they allow the monitoring of incidence and mortality trends in large patient populations.

Important changes in the management of septic patients have been included in recent years. Strategies to increase awareness in healthcare workers [9, 10], emphasis on the achievement of early empiric effective antibiotic treatment [9–11], simplification of the hemodynamic management (early goal directed therapy [12] versus last evidence [13–15]), changes on respiratory failure management (use of prone positioning [16], protective ventilation [17, 18], ECMO [19, 20]), or renal failure management [21] presented some differences during the last years. However, there is no information about how changes in the management of sepsis-associated organ failure have contributed to mortality reduction during these years.

The objective of our study is to observe trends in mortality in septic patients according to the different organ failure in the population of Catalonia over a period of 15 years.

## Material and methods

### Data sources

A retrospective analysis was conducted from hospital discharge records from the Minimum Basic Data Set Acute-Care Hospitals (CMBD-HA in Catalan language) for the Catalan Health System (CatSalut). The study was approved by the Mataró’s Hospital and Maresme Health Consortium Ethical Review Board with a waiver of informed consent in 21th of March in 2018 (code CEIC_20/18), and all study’s procedures were followed in accordance with the Helsinki Declaration of 1975.

CMBD-HA is a mandatory population-based register of admissions to all public and private acute-care hospitals in Catalonia that enables evaluation and optimization of resource use, provides support, improves healthcare planning and facilitates procurement management and payments. All the codes are provided directly by the patient's treating physicians and subsequently verified by the technical secretariat of each health center. To ensure data quality, the CMBD-HA input data are systematically validated internally in CatSalut with an automated data validation system that checks data consistency and identifies potential errors or inconsistencies between variables. Furthermore, as this information is used for provider payment purposes, external audits are regularly performed to ensure the quality and reliability of the data. These external audits are performed whenever a suspicious deviation is detected or, if none is detected, every 3 or 5 years. The data set contains demographic and clinical data for patient care episodes, including age, sex, length of stay (days), one primary diagnosis, up to fourteen secondary diagnoses, one primary procedure, up to nineteen secondary procedures and status on discharge (alive, dead, or transferred to another hospital). Official data from the register of insured persons maintained by CatSalut were used to estimate crude and specific hospitalization rates (universal coverage for 7.570.430 inhabitants in 2019).

### Patients

Sepsis was defined by the presence of infection and at least one organ dysfunction. In Catalonia, the diagnostic coding system changed in January 2018 from ICD-9-CM to ICD-10-CM. In this way, ICD-9-CM codes were used until December 31, 2017, and ICD-10-CM were used subsequently.

Using ICD-9-CM and ICD-10-CM codes, all hospitalized patients with and infection and an organ dysfunction were detected following the Angus methodology [22], over a 15-year period (2005–2019). All ICD-9-CM diagnostic codes used for detection of infection-related organ dysfunction have been provided in the Additional file 1, and refer to acute dysfunction of any organ as a result of sepsis, since each of them is associated with the diagnosis of infection or sepsis. All of these used codes were translated into the new coding system (CID-10-CM) as of 2018.

To avoid overlaps, we excluded patients who were transferred from one acute-care hospital to another during the same sepsis episode; 18.992 admissions were excluded from non-residents in Catalonia.

### Coding

Diagnoses and procedures were coded using the ICD-9-CM until the end of 2017 and ICD-10-CM for 2018–2019. ICD-9-CM codes to identify patients with sepsis were updated in 2000 to the following: 995.91 (sepsis), 995.92 (severe sepsis) and 785.52 (septic shock). ICD-10-CM codes to identify patients with sepsis were equated to ICD-9-CM codes and were: R652 (severe sepsis), R6520 (severe sepsis without septic shock) and R6521 (severe sepsis with septic shock).

Although information was not available regarding the unit or department where patients were treated (intensive care unit (ICU), internal medicine unit, etc.), we indirectly deduced ICU admission from procedures typically used in intensive care management (Additional file 1). The Charlson comorbidity index with its 17 comorbid disease categories [23] was used to assess the presence of underlying comorbidities. The ICD-9-CM codes used to identify acute organ dysfunction and infectious processes are listed in Supplementary Material. With ICD-10-CM codification system, all used codes to identify acute organ dysfunction were equated to ICD-10-CM system.

### Statistical analysis

The hospitalization rate was defined as the yearly number of admissions per 100.000 population. Crude overall and specific hospitalization rates by age and sex were calculated. Continuous variables and discrete variables were compared using analysis of variance and the Chi-square test, respectively. Multivariate logistic regression, adjusted for other significant variables, was used to analyze hospital mortality risk by year of admission for the study population and for the ICU and non-ICU patient groups; variables were entered one by one and retained when their significance was < 0.10 and were clinically plausible. For the regression analysis, each of the clinical attributes included (comorbidities, acute organ failure and infection) was treated as binary (dummy) variables indicating the presence or absence of these conditions; a single patient could therefore account for more than one attribute. The area under the receiver operating characteristic curve (AUROC) was used to evaluate how well the multivariate logistic regression model discriminated between patients with severe sepsis who were discharged alive versus those who died in hospital [24]. Data analysis was performed using SPSS 18.0 software (SPSS Inc, Chicago, IL, USA).

## Results

Of 11.916.974 discharges from all acute-care hospitals during the study period (2005–2019), 296.554 had sepsis (2.49%). Demographic characteristics and comorbidities for patients with sepsis are shown in Table 1.

**Table 1:** Profile of patients with severe sepsis in Catalonia

	Total N = 296,554	Alive N = 238,775	Dead N = 57,779
	N (%)	N (%)	N (%)
Sex (males)	166,808 (56.2%)	133,428 (55.9%)	33,380 (57.8%)

	Mean (SD)	Mean (SD)	Mean (SD)
Charlson index	5.51 (2.74)	5.37 (2.72)	6.08 (2.74)
Mean age	72.9 (18.1)	72.3 (18.7)	75.2 (15.3)
Length of hospital stay (days)	15.3 (24.2)	15.5 (24.8)	14.5 (21.6)
*Age (years)*			
< 15	6430 (2.17%)	5905 (2.47%)	525 (0.91%)
15–44	15,072 (5.08%)	13,332 (5.58%)	1740 (3.01%)
45–64	47,027 (15.9%)	38,020 (15.9%)	9007 (15.6%)
65–74	53,585 (18.1%)	42,924 (18.0%)	10,661 (18.5%)
75–84	94,322 (31.8%)	75,577 (31.7%)	18,745 (32.4%)
> 84	80,118 (27.0%)	63,017 (26.4%)	17,101 (29.6%)
*Comorbidities*			
Myocardial infarction	12,377 (4.17%)	9481 (3.97%)	2896 (5.01%)
Congestive heart failure	65,456 (22.1%)	50,537 (21.2%)	14,919 (25.8%)
Peripheral vascular disease	16,134 (5.44%)	12,452 (5.21%)	3682 (6.37%)
Cerebrovascular disease	22,538 (7.60%)	17,568 (7.36%)	4970 (8.60%)
Dementia	23,510 (7.93%)	18,791 (7.87%)	4719 (8.17%)
COPD	74,240 (25.0%)	60,892 (25.5%)	13,348 (23.1%)
Rheumatic diseases	6952 (2.34%)	5801 (2.43%)	1151 (1.99%)
Peptic ulcer disease	3512 (1.18%)	2666 (1.12%)	846 (1.46%)
Liver disease: mild	29,962 (10.1%)	22,517 (9.43%)	7445 (12.9%)
Uncomplicated diabetes	64,848 (21.9%)	54,717 (22.9%)	10,131 (17.5%)
Complicated diabetes	14,909 (5.03%)	12,689 (5.31%)	2220 (3.84%)
Hemiplegia/paraplegia	4896 (1.65%)	3966 (1.66%)	930 (1.61%)
Chronic kidney disease	83,949 (28.3%)	69,525 (29.1%)	14,424 (25.0%)
Cancer	41,129 (13.9%)	29,039 (12.2%)	12,090 (20.9%)
Liver disease: moderate–severe	9121 (3.08%)	6291 (2.63%)	2830 (4.90%)
Metastasis	15,473 (5.22%)	9952 (4.17%)	5521 (9.56%)
AIDS/HIV infection	2781 (0.94%)	2113 (0.88%)	668 (1.16%)
ICU admissions*	70,578 (23.8%)	46,354 (19.4%)	24,224 (41.9%)
*Sepsis origins*			
Urinary	113,565 (38.3%)	99,236 (41.6%)	14,329 (24.8%)
Respiratory	96,940 (32.7%)	76,410 (32.0%)	20,530 (35.5%)
Abdominal	31,518 (10.6%)	24,001 (10.1%)	7517 (13.0%)
Skin and soft tissues	13,465 (4.54%)	10,985 (4.60%)	2480 (4.29%)
Endocarditis	3789 (1.28%)	2712 (1.14%)	1077 (1.86%)
Device related	22,063 (7.44%)	17,968 (7.53%)	4095 (7.09%)
CNS	2457 (0.83%)	1849 (0.77%)	608 (1.05%)
Others	9537 (3.22%)	8175 (3.42%)	1362 (2.36%)
Unspecific	40,578 (13.7%)	27,231 (11.4%)	13,347 (23.1%)
Bacteremia	84,191 (28.4%)	56,291 (23.6%)	27,900 (48.3%)
	Total N = 296,554	Alive	Dead
*Organ Dysfunction*			
Kidney	168,603 (56.9%)	135,059 (56.6%)	33,544 (58.1%)
Lung	45,016 (15.2%)	28,734 (12.0%)	16,282 (28.2%)
CNS	61,377 (20.7%)	53,209 (22.3%)	8168 (14.1%)
Hematologic	30,615 (10.3%)	24,547 (10.3%)	6068 (10.5%)
Cardiovascular	71,929 (24.3%)	46,306 (19.4%)	25,623 (44.3%)
Liver	3956 (1.33%)	1819 (0.76%)	2137 (3.70%)
*Number of organ failures*			
1	231,622 (78.1%)	197,135 (82.6%)	34,487 (59.7%)
2	48,898 (16.5%)	33,865 (14.2%)	15,033 (26.0%)
3 or more	16,034 (5.41%)	7775 (3.26%)	8259 (14.3%)

Data are presented as mean and standard deviation or %

*AIDS*: acquired immune deficiency syndrome, *CNS:* central nervous system, *COPD:* chronic obstructive pulmonary disease, *HIV:* human immunodeficiency virus, *ICU:* intensive care unit

ICU admissions* are estimated from invasive procedures related to organ failure management

The annual sepsis incidence in the population was 264.1 per 100.000 inhabitants/year, and it increased every year, going from 144.5 in 2005 to 410.1 in 2019.

Sepsis was significantly associated with age (76.9% of the cases occurred in patients older than 65 years) and sex (56.2 of cases occurred in men) (*p* < 0.001).

**Fig. 1:**
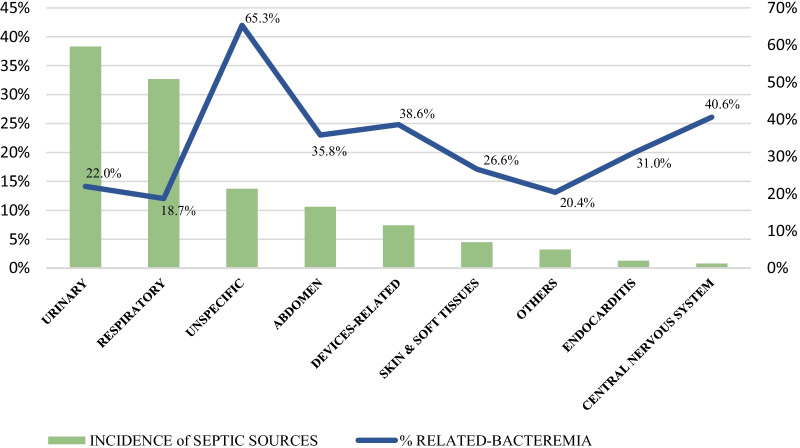
Incidence of different septic sources and its related-bacteremia

The most frequent origins of sepsis were urinary and respiratory tract infections, accounting for 38.3% and 32.7% of cases, respectively, followed by sepsis of unknown origin (13.7%). The central nervous system was the least frequent origin of sepsis (0.8%) (Fig. 1).

More than a quarter of cases (26.3%) presented bacteremia. Infections with most presence of bacteremia were those related to unknown origin (65.3%), followed by central nervous system (40.6%), external devices (38, 6%) and abdominal sources (35.8%). The source with the lowest presence of bacteremia was the respiratory tract (18.7%) (Fig. 1).

**Fig. 2:**
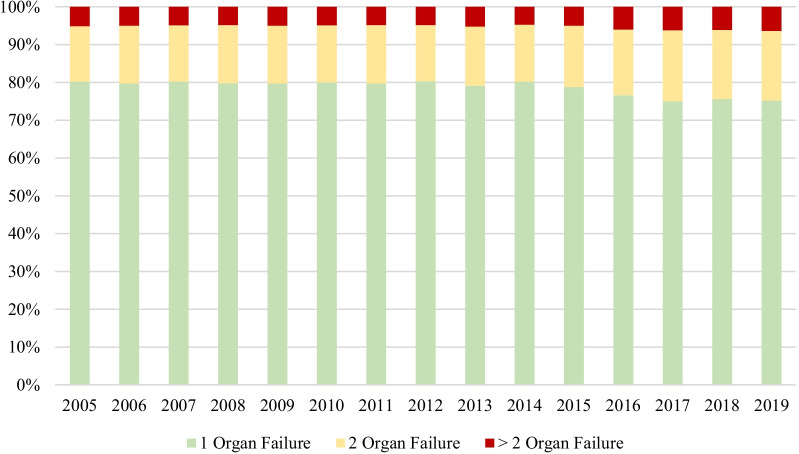
Incidence of 1, 2 or ≥ 2 organ failures in sepsis during study period (2005 – 2019)

The majority of patients (78.1%) presented single organ failure at the time of sepsis diagnosis and slightly more than 5% had failure of 3 or more organs in this moment (Table 1, Fig. 2).

**Fig. 3:**
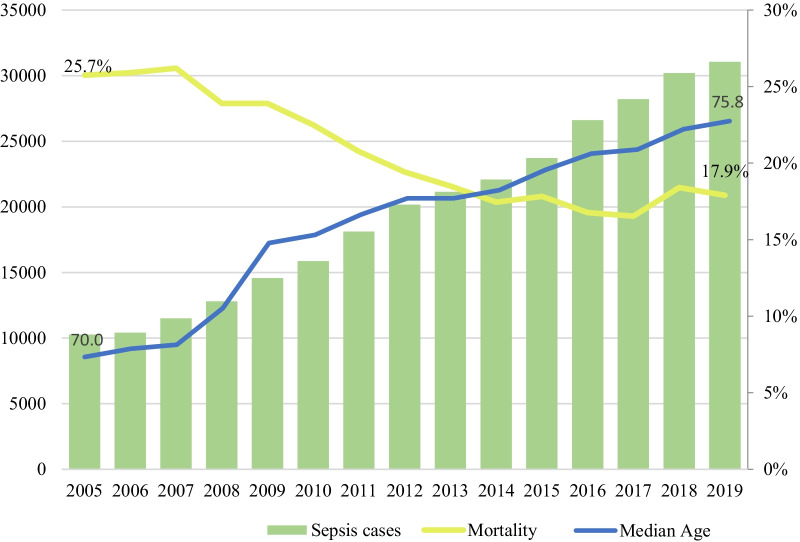
Incidence, mortality and median age of Sepsis in Catalonia during study period (2005–2019)

Global length of hospital stay was 15.3 (± 24.2) days, and it was shorter in those patients who died (14.5 (± 21.6) vs 15.5 (± 24.8). Length of hospital stay has been decreasing from 19.5 (± 25.4) days in 2005 to 14.9 (± 21.4) days in 2019 (*p* < 0.0001). Hospital mortality during the study period was 19.5% but decreases continuously from 25.7% in 2005 to 17.9% in 2019 (*p* < 0.0001) (Fig. 3). Hospital mortality was higher in older patients (*p* < 0.05) and in those cases that presented some comorbidity included in the Charlson index assessment (*p* < 0.0001) (Table 1).

### Organ failure-associated mortality

Renal was the most frequent organ failure (56.9%), followed by cardiovascular (24.3%), central nervous system (20.7%) and respiratory failure (15.2%). Hepatic failure was the less frequent organ failure present in septic patients (1.3%) (Table 1).

**Fig.4:**
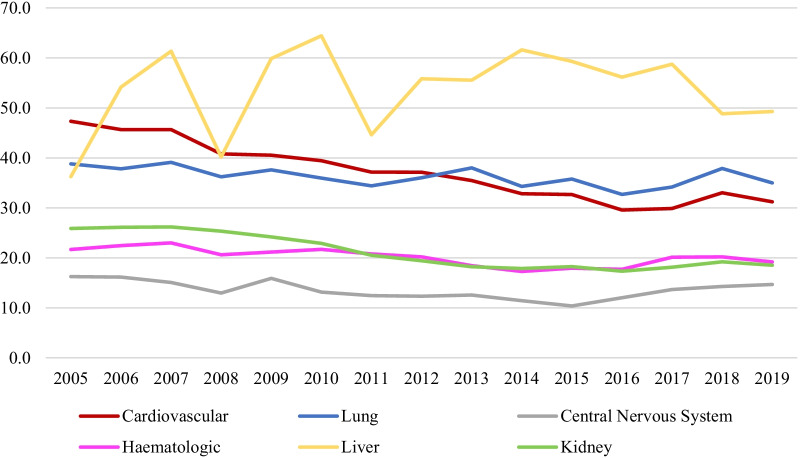
Evolution of mortality (%) in sepsis according to organic dysfunction

Sepsis-related cardiovascular failure mortality was 35,6% during the study period. However, a reduction in this rate was observed in recent years (from 47.3% in 2005 to 31.2% in 2019) (*p* < 0.0001) (Fig. 4). In the same way, mean mortality related to renal failure in sepsis was 19.9%, but a reduction in this mortality was observed in last years (from 25.9% in 2005 to 18.5% in 2019) (*p* < 0.0001) (Fig. 4). In relation to respiratory failure, mortality also declined in last period from 38.8% in 2005 to 35% in 2019 (*p* < 0.0001), as well as in central nervous system failure (from 16.3% in 2005 to 14.7% in 2019, *p* < 0.0001) and hematological failure (from 21.7% in 2005 to 19.2% in 2019, *p* < 0.0001) (Fig. 4).

Although hepatic failure is the least frequent organic failure in sepsis, mortality in these patients was high (52.0%) and has been increasing over the years (from 36.3% in 2005 to 49.3% in 2019, *p* < 0.0001) (Fig. 4). These differences were confirmed in the multivariate analysis adjusted for all significant variables (age group, sex, comorbidities, organ dysfunction, infection source and presence of bacteremia) (Table 2).

**Table 2:** Univariate and multivariate analyses of organ failure associated in-hospital mortality by year of admission in Catalonia (2005–2017)

Univariate analysis				Multivariate analysis		
Year	n	Mortality	*p*	OR	IC95%	*p*
*Respiratory failure*						
2005	2662	38,8		1	–	
2006	2459	37,8		0,8,555,715	0.755–0.970	0.0146
2007	2643	39,2		0,89,399,059	0.791–1.011	0.0731
2008	2799	36,2		0,77,898,524	0.690–0.880	< 0.0001
2009	2996	37,6		0,75,801,748	0.673–0.854	< 0.0001
2010	2816	36,0		0,69,558,657	0.616–0.786	< 0.0001
2011	2802	34,4		0,60,252,572	0.533–0.681	< 0.0001
2012	2776	36,0		0,58,824,668	0.520–0.665	< 0.0001
2013	2891	38,0		0,61,456,548	0.545–0.693	< 0.0001
2014	2642	34,3		0,51,394,317	0.453–0.583	< 0.0001
2015	2745	35,8		0,52,879,873	0.468–0.598	< 0.0001
2016	3380	32,7		0,46,018,306	0.408–0.518	< 0.0001
2017	3849	34,3		0,48,323,359	0.431–0.542	< 0.0001
2018	3742	37,9	< 0.0001	0,56,156,227	0.501–0.630	< 0.0001
2019	3814	35,0		0,4,733,619	0.422–0.531	< 0.0001
					AUC ROC	
					0.762 (0.758 – 0.767)	
*Cardiovascular failure*						
2005	2491	47,3		1	–	
2006	2858	45,7		0,69,393,529	0.615–0.783	< 0.0001
2007	3225	45,7		0,69,297,304	0.616–0.779	< 0.0001
2008	3568	40,8		0,55,888,678	0.498–0.627	< 0.0001
2009	3945	40,5		0,54,844,547	0.490–0.614	< 0.0001
2010	4081	39,4		0,48,303,417	0.431–0.541	< 0.0001
2011	4487	37,1		0,44,050,755	0.394–0.493	< 0.0001
2012	4520	37,1		0,40,863,827	0.365–0.457	< 0.0001
2013	4774	35,5		0,37,430,685	0.335–0.418	< 0.0001
2014	4802	32,8		0,33,977,245	0.304–0.380	< 0.0001
2015	5256	32,7		0,32,401,597	0.290–0.362	< 0.0001
2016	6389	29,6		0,27,741,318	0.249–0.309	< 0.0001
2017	5791	29,9		0,25,132,744	0.225–0.281	< 0.0001
2018	7504	33,0	< 0.0001	0,32,937,548	0.297–0.366	< 0.0001
2019	8238	31,2		0,3,003,618	0.271–0.333	< 0.0001
					AUC ROC	
					0.764 (0.761 – 0.768)	
*CNS failure*						
2005	1809	16,2		1	–	
2006	1676	16,2		1,003,113,881	0.827–1.217	0.9749
2007	2036	15,1		0,984,914,164	0.818–1.186	0.8727
2008	2392	12,9		0,783,057,153	0.651–0.941	0.0092
2009	2648	15,9		0,983,827,452	0.826–1.171	0.8547
2010	2865	13,1		0,821,556,085	0.689–0.980	0.0292
2011	3197	12,4		0,727,698,472	0.611–0.866	0.0003
2012	3860	12,3		0,676,469,996	0.571–0.801	< 0.0001
2013	3855	12,5		0,703,124,481	0.594–0.832	< 0.0001
2014	4237	11,4		0,65,610,759	0.555–0.776	< 0.0001
2015	4704	10,4		0,55,191,349	0.467–0.652	< 0.0001
2016	6238	12,0		0,569,267,239	0.486–0.667	< 0.0001
2017	7081	13,7		0,624,865,732	0.536–0.729	< 0.0001
2018	7542	14,2	< 0.0001	0,673,377,407	0.578–0.784	< 0.0001
2019	7237	14,7		0,64,039,723	0.549–0.746	< 0.0001
					AUC ROC	
					0.761 (0.755 – 0.766)	
*Hematological failure*						
2005	1089	21,7		1	–	
2006	1122	22,4		1,02,282,062	0.808–1.295	0.8513
2007	1218	23,0		1,03,249,027	0.819–1.301	0.7866
2008	1304	20,6		0,85,500,995	0.679–1.077	0.1829
2009	1578	21,2		0,80,145,653	0.643–0.999	0.0492
2010	1705	21,7		0,81,385,536	0.655–1.011	0.0625
2011	1814	20,8		0,7,338,104	0.592–0.910	0.0048
2012	1931	20,2		0,67,176,921	0.542–0.832	0.0003
2013	2141	18,4		0,56,482,716	0.457–0.698	< 0.0001
2014	2060	17,3		0,52,934,679	0.426–0.657	< 0.0001
2015	2239	18,0		0,56,450,526	0.457–0.697	< 0.0001
2016	2822	17,7		0,4,413,267	0.360–0.542	< 0.0001
2017	2883	20,3		0,45,095,202	0.368–0.552	< 0.0001
2018	3327	20,2	< 0.0001	0,529,861	0.435–0.646	< 0.0001
2019	3382	19,2		0,47,667,204	0.391–0.581	< 0.0001
					AUC ROC	
					0.835 (0.829 – 0.840)	
*Liver failure*						
2005	80	36,2		1	–	
2006	96	54,2		1,83,497,874	0.892–3.776	0.0992
2007	119	61,3		1,96,503,495	0.995–3.882	0.0518
2008	107	40,2		0,85,993,432	0.430–1.721	0.6698
2009	147	59,9		2,07,901,315	1.072–4.032	0.0304
2010	149	64,4		1,93,607,915	0.998–3.754	0.0505
2011	186	44,6		0,95,603,365	0.506–1.805	0.8898
2012	240	55,8		1,35,968,041	0.736–2.513	0.3269
2013	252	55,6		1,31,101,581	0.712–2.415	0.3848
2014	250	61,6		1,31,593,221	0.712–2.434	0.3815
2015	371	59,3		1,22,325,659	0.678–2.208	0.5035
2016	365	56,2		0,97,385,455	0.540–1.756	0.9298
2017	382	58,9		0,96,491,164	0.534–1.744	0.9059
2018	514	48,8	< 0.0001	0,72,797,746	0.400–1.326	0.2992
2019	698	49,3		0,68,868,416	0.383–1.239	0.2134
					AUC ROC	
					0.801 (0.788 – 0.815)	
*Kidney failure*						
2005	4780	25,9		1	–	
2006	4937	26,1		0,940,454,002	0.850–1.040	0.2332
2007	5195	26,2		0,923,138,453	0.836–1.020	0.1160
2008	5930	25,3		0,873,826,895	0.793–0.963	0.0065
2009	7090	24,2		0,830,444,748	0.756–0.912	0.0001
2010	8326	22,9		0,758,868,867	0.692–0.832	< 0.0001
2011	10,341	20,5		0,659,592,374	0.603–0.721	< 0.0001
2012	12,025	19,4		0,609,565,863	0.558–0.665	< 0.0001
2013	12,980	18,2		0,53,201,268	0.488–0.580	< 0.0001
2014	13,721	17,9		0,535,202,205	0.491–0.584	< 0.0001
2015	14,896	18,2		0,531,784,828	0.488–0.579	< 0.0001
2016	15,659	17,3		0,456,185,713	0.419–0.497	< 0.0001
2017	17,538	18,1		0,465,317,937	0.428–0.506	< 0.0001
2018	17,311	19,2	< 0.0001	0,511,196,703	0.470–0.556	< 0.0001
2019	17,874	18,5		0,45,881,633	0.422–0.499	< 0.0001
					AUC ROC	
					0.783 (0.780 – 0.786)	

Data are presented as number of death or %. The multivariate analysis is adjusted by sex, age group, comorbidities, ICU admission, emergency admission, organ dysfunction, number of organ failures, sepsis origin and bacteremia

*CI* confidence interval, *ICU* intensive care unit, *OR* odds ratio, *NS* non-significant

## Discussion

This observational study shows that the association of organ failure with mortality has changed over time depending on the affected organ. To our knowledge, there are no epidemiological studies that have analyzed the evolution of the behavior of mortality associated with the different organ failures in septic patients.

Protocols to increase the detection of sepsis [25], better antimicrobial stewardship [26] and initiate early source control [27] have led to an improvement in the vital prognosis of patients with multiorgan failure. For this reason, an improvement in survival of all organ failures analyzed separately would be expected. Nevertheless, our study shows that this impact is not homogeneous. Although the nature of our study does not allow us to establish causal relationships, we suggest that the differences in the evolution of mortality associated with each organ failure could be related to an improvement in the care of some of them (cardiovascular failure) compared to those without specific treatment (liver failure). However, studies designed for this purpose should be developed to confirm this hypothesis.

In our opinion, the most relevant result in our analysis is the reduction in mortality in the cardiovascular failure group. The continuous reduction observed, from 47.3 to 31.2% of mortality, supposes about a one third relative reduction in mortality. Evolution in management protocols has greatly simplified the initial management of septic shock. Current protocols advocate for a lower positive fluid balance [28, 29] and an early use of norepinephrine [30, 31], which allows an earlier recovery of tissue perfusion [32, 33].

We do not believe that the reduction in mortality in renal failure could be due to an improvement in extrarenal clearance techniques, the lack of consensus on which is the best modality or the moment of initiation of the technique may hinder a greater impact [34]. However, the close relationship between the improvement in tissue perfusion and renal function is well known, which could explain the parallelism between improved cardiovascular and renal failure survival.

There is also a reduction in mortality in respiratory failure, although not so marked. Although non-invasive techniques (high-flow nasal cannulas, non-invasive mechanical ventilation) [35, 36] have failed to significantly impact the general prognosis of patients with sepsis, in some subpopulations they do appear to be useful. The incorporation of recruitment maneuvers (prone position, PEEP,) and the use of extracorporeal techniques can also explain this better prognosis.

Improvement in each organ failure mortality rates results in a reduction in global mortality on septic patients. The general trend observed in our study is also present in other observational studies, both in the epidemiological characteristics of the patients and in the origin and impact of infections [3, 4, 22, 37–40]. Liver failure, however, presents an opposite trend.

Sepsis is not considered in epidemiological studies as a major cause of acute liver failure [41]. However, when it appears, it defines a scenario of high mortality. Liver failure in sepsis does not have specific treatment or organ support measures. The use of extracorporeal techniques in sepsis for liver support is still anecdotic and cannot be considered a standardized technique [42]. Macrophage activation-like syndrome (MALS) in septic patients causes hepatic dysfunction and hematological alterations and, when present, significantly increases mortality in these patients [43]. MALS, which does not respond to standard sepsis treatment, could explain the high mortality of septic patients with liver failure and the lack of prognostic improvement that septic patients with hematological dysfunction have experienced over the years.

Knowing the dimensions of sepsis at the population level is essential for a rational use of economic and health resources. The incidence of sepsis increases year after year, and mortality has been decreasing in parallel. Our data are consistent with other epidemiological studies both in Europe [3, 39, 40] and in other settings [4, 22, 37, 38] and also with clinical data from population studies in our territory [8]. The increase in incidence is attributed to a better control of other pathologies, increase in life expectancy and increase in patient’s age [44] (Fig. 3), though it should be noted that an increase in diagnostic coding in recent years could have contributed to the progressive increase in the incidence of sepsis.

As mentioned above, population-based epidemiological studies such as ours do not allow causality to be established, but the improvement in the results could be attributed to the improvement in the knowledge of the physiopathology of sepsis and the improvement and standardization of treatments, related to the implementation of action plans to improve septic patient care. Since the beginning of 2015, the Interhospital Sepsis Code (CSI) has existed in Catalonia. The CSI is a territorial strategy that involves all the CatSalut acute hospitals and defines an emergency care plan for patients with sepsis; its objective is to speed up detection and coordinate care between hospitals of different complexity at the territorial level throughout the country [45].

Our study has been carried out in the population of Catalonia. Although other studies have shown results similar to ours [3, 4, 22, 37–40], this marks a limitation that must be considered since the results may not be extrapolated to other countries with different socioeconomic levels or dissimilar health systems. Admission to the ICU was deduced by procedure coding and, although in our environment the procedures related to critical or semi-critical patients are performed mostly in intensive care units, this does not happen in 100% of cases. For this reason, the percentage of patients admitted to the ICU could be overestimated as a result of selection bias.

The most relevant limitation of this study is that it is a retrospective epidemiological study based on hospital discharge data. Although we have used a validated methodology for the case definition, there is a risk of bias related to inconsistency in the definition of the processes and in the coding. Despite that the clinical diagnostic criteria for sepsis were modified and updated during the study period [46] (a fact that may limit homogeneity when identifying cases), infection and organ failure definitions remained unchanged, so inclusion criteria are unmodified. On the other hand, codification system was changed in Catalonia in the beginning of 2018 from ICD-9-CM to ICD-10-CM. This change could be the responsible for the slight increase in mortality during 2018 and 2019 compared to previous years although this does not affect the organ failure associated trend.

We would like also comment that we excluded the pandemic period from the analysis. It is unknown how could affect sepsis prognosis the impact of changes in hospital’s structures, the effect of limited human and structural resources to attend critically ill patients or the impact of social restrictions during the pandemic. In our opinion, it requires an specific and very interesting analysis.

## Conclusions

The incidence of sepsis has been increasing in recent years in our setting. However, hospital mortality has been significantly reduced. In septic patients, all organ failures except liver have shown a statistically significant reduction on associated mortality, with cardiovascular failure as the most relevant. Early source control and the simplification of algorithms to recover tissue perfusion could explain these results. On the contrary, mortality associated with liver failure in sepsis is very high and has not changed, a fact that could be explained by the lack of specific treatment for the failure of this particular organ.

## Supplementary Information

**Additional file 1. **ICD‑9‑MC codes used to identify infectious process, site of infection, acute organ dysfunction and procedures used in intensive care unit.

## Data Availability

The datasets used and analyzed during the current study are available from the corresponding author on reasonable request.
